# CDCA gene family promotes progression and prognosis in lung adenocarcinoma

**DOI:** 10.1097/MD.0000000000038581

**Published:** 2024-06-14

**Authors:** XiangSen Liu, Xudong Zhu, Yi Zhao, Yuchen Shan, ZhaoJia Gao, Kai Yuan

**Affiliations:** a Department of Thoracic Surgery, The Affiliated Changzhou No. 2 People’s Hospital of Nanjing Medical University, Changzhou, China; b Heart and Lung Disease Laboratory, The Affiliated Changzhou No. 2 People’s Hospital of Nanjing Medical University, Changzhou, China.

**Keywords:** CDCA, cell cycle, prognostic value, tumor biomarker, gene family, lung adenocarcionma

## Abstract

**Background:**

The cell division cycle-associated (CDCA) family participates in the cell cycle, and the dysregulation of its expression is associated with the development of several types of cancers. However, the roles of CDCAs in lung adenocarcinomas (LUAD) have not been investigated in systematic research.

**Methods:**

Using data retrieved from The Cancer Genome Atlas (TCGA), the expression of CDCAs in LUAD and normal tissues was compared, and survival analysis was performed using the data. Also, the correlation between clinical characteristics and the expression of CDCAs was assessed. Using data from cBioPortal, we investigated genetic alterations in CDCAs and their prognostic implications. Immunohistochemical analyses were performed to validate our findings from TCGA data. Following this, we created a risk score model to develop a nomogram. We also performed gene set enrichment analyses (GSEA), gene ontology, and KEGG pathway analysis. We used Timer to analyze the correlation between immune cell infiltration, tumor purity, and expression data.

**Results:**

Our results indicated that all CDCAs were expressed at high levels in LUAD; this could be associated with poor overall survival, as indicated in TCGA data. Univariate and multivariate Cox analyses revealed that CDCA4/5 could serve as independent risk factors. The results of immunohistochemical analyses confirmed our results. Based on the estimation of expression levels, clinical characteristics, alterations, and immune infiltration, the low-risk group of CDCA4/5 had a better prognosis than the high-risk group. Immune therapy is also a potential treatment option.

**Conclusion:**

In conclusion, our findings indicate that CDCAs play important roles in LUAD, and CDCA4/5 can serve as diagnostic and prognostic biomarkers and therapeutic targets in LUAD.

## 1. Introduction

According to global cancer statistics, in 2020,lung cancer was the primary cause of cancer deaths, attributed in an estimated 1.8 million deaths (18%).^[1]^ A potential reason could be that the condition is often diagnosed in the late stages, and patients do not exhibit obvious symptoms at an early-stage.^[2]^ Non-small cell lung cancer (NSCLC) accounts for 85% of lung cancer cases, and lung adenocarcinomas (LUAD) accounts for approximately 40% of NSCLC cases.^[3]^ Early diagnosis is of great significance in patients because the their prognosis with cancer at different clinical stages is considerably different. For example, the 5-year survival rate of patients with stage IA can exceed 90%, whereas the survival rate of patients with stage IV is slightly <10%.^[4]^ LUAD is associated with a high incidence and poor prognoses; hence, early diagnosis is of great importance to patients with LUAD. Currently, imaging examinations and pathological biopsy are the primary diagnostic methods; however, these methods cannot be used to diagnose early-stage LUAD as they are not sufficiently advanced.^[5]^ Therefore, identifying tumor biomarkers is crucial for the early detection and improvement of prognosis in patients with LUAD.

Cell division cycle-associated (CDCA) gene family comprises 8 members, namely CDCA1-8. CDCA genes contribute to cell cycle regulation.^[6]^ The cell division cycle plays an important role in the life process, and the its dysregulation is often related to cancer.^[7,8]^ Nowadays, more studies are being conducted on CDCA genes as prognostic factors in cancers such as nasopharyngeal carcinoma, hepatocellular carcinoma, ovarian cancer, and prostate cancer.^[9–12]^ However, the role of CDCA in patients with LUAD has not been analyzed. CDCA1, also known as NUF2, is a member of the centromere protein complex that is evolutionarily conserved. CDCA1 is a cancer assessment antigen, and its upregulation is a common and important characteristic of cancer cell growth and survival^.[13]^ CDCA2 expression is significantly correlated with the expression of components related to cell cycle phase transition, and CDCA2 overexpression plays a significant role in tumorigenesis.^[14]^ By stimulating certain proteins, cell mitosis and some physiological and pathological processes are regulated by CDCA3. In addition, CDCA3 is a potential prognostic biomarker for various cancers.^[15]^ CDCA4 participates in the cell proliferation through the E2F/retinoblastoma protein pathway and its expression is induced when cells enter the G1/S phase.^[16]^ CDCA5 is a substrate of the anaphase-promoting complex. It has been reported to be overexpressed in multiple cancers, and it promoted cell proliferation by activating the PI3K/AKT/mTOR pathway.^[17]^ CDCA6, also called CBX2, has been shown to shape chromatin accessibility to promote AML via the p38 MAPK signaling pathway.^[18]^ It regulates proliferation and apoptosis by activating the phosphorylation of YAP.^[19]^ CDCA7 expression is related to the migration and invasion of lymphoma,^[20]^ and patients with CDCA7 overexpression have poorer prognosis in breast cancer.^[21]^ CDCA8 also promotes the development of live cancer^[22]^ and pancreatic cancer.^[23]^

In this study, we intended to: Investigate the relationship between the expression of the CDCAs and clinical characteristics of patients with LUAD; analyze whether the CDCAs could serve as diagnostic and prognostic biomarkers in LUAD; evaluate the expression model of CDCAs to predict the survival time of individual patients; explore the known mechanisms using bioinformatics analysis.

## 2. Materials and methods

### 2.1. Acquisition of patient mRNA expression data

We retrieved the mRNA expression data from the the cancer genome atlas (TCGA) database (https://cancergenome.nih.gov/) in February 2023.^[24]^ The differential expression patterns of CDCA proteins in tumor (n = 541) and normal tissues (n = 59) were compared using TCGA.

### 2.2. Correlation of CDCA expression patterns with clinical characteristics in LUAD

The correlation of CDCA expression patterns with the different clinical characteristics (LUAD: age, ≤65, n = 239; > 65, n = 258; gender, female, n = 272, male, n = 235; Stage, Stage I–II, n = 392; Stage III–IV, n = 107; N, NO = 327, N1-3, n = 168) was analyzed using R. We used Wilcoxon test to analyze the correlation between the expression level of CDCA and the clinical characteristics of LUAD.

### 2.3. Alterations in CDCA expression patterns and their prognostic value

First, 4 datasets (n = 1658) (LUAD: MSK, J Thorac Oncol 2020, Broad, Cell 2012, OncoSG, Nat Genet 2020, TCGA, PanCancer Atlas) were selected from cBioPortal (http://www.cbioportal.org/), a public website for exploring cancer genomics^[25]^ to analyze the type and proportion of genetic alterations in the CDCAs. Following this, Kaplan–Meier (KM) analysis was used to analyze the association between alteration and overall survival (OS) in LUAD. Patients with LUAD were divided into 2 groups (with or without the genetic alterations), and we use the log-rank test to compare the differences between the survival rates in the 2 groups.

### 2.4. Prognostic value of CDCAs

By analyzing the survival information from TCGA (retrieved in February 2023; n = 512), we estimated the prognostic value of CDCA gene expression. After excluding patients with missing data, 507 patients were included in the study (Table 1). Based on mRNA expression patterns, we categorized patients with LUAD into high-expression and low-expression groups and analyzed the overall survival rate using the KM survival curve. Furthermore, we used univariate and multivariate Cox analysis to explore the prognostic value and potential as independent prognostic factors. The difference was considered to be statistically significant at *P*-values < .05.

**Table 1:** 507 LUAD patients in TCGA database.

Characteristic	Levels (n = 507)	Overall
Gender, n (%)	Female	272 (53.6%)
	Male	235 (46.4%)
	Unknown	0 (0%)
Age, n (%)	<=65	239 (47.1%)
	>65	258 (50.9%)
	Unknown	10 (2.0%)
Pathologic stage, n (%)	Stage I	272 (53.6%)
	Stage II	120 (23.7%)
	Stage III	81 (16.0%)
	Stage IV	26 (5.1%)
	Unknown	8 (1.6%)
T stage, n (%)	T1	169 (33.3%)
	T2	271 (53.5%)
	T3	45 (8.9%)
	T4	19 (3.7%)
	Unknown	3 (0.6%)
N stage, n (%)	N0	327 (64.5%)
	N1	95 (18.7%)
	N2	71 (14.0%)
	N3	2 (0.4%)
	Unknown	12 (2.4%)
M stage, n (%)	M0	338 (66.7%)
	M1	25 (4.9%)
	Unknown	144 (28.4%)
OS event, n (%)	Alive	324 (63.9%)
	Dead	183 (36.1%)
	Unknown	0 (0%)

### 2.5. Risk score model for nomogram

Based on the expression patterns of CDCA4/5 genes, a risk score model was created. The risk score was calculated using the following formula: $Risk\;score=\sum_{i=1}^{n}coefi\times Xi$. The “coefi” refers to the coefficient, whereas “Xi” refers to the expression of selected genes. Based on our risk score result, we categorized half of the samples from TCGA in the high-risk group and the remaining in the low-risk group. The difference between the overall survival rates in the 2 groups is analyzed using the log-rank test. The “survivalROC” package was installed to conduct receiver operating characteristic (ROC) curve analysis and estimated the reliability of risk score using the area under the curve (AUC) values. We also performed univariate and multivariate Cox analyses to explore whether our risk score could be considered an independent prognostic factor if we combined other factors like sex, age, and T, N, M and stages. Finally, we created a nomogram to predict the survival duration of different patients based on the result of univariate and multivariate Cox analyses.

### 2.6. Tissue microarray and analysis of immunohistochemical results

To confirm our findings from the analysis of TCGA data, we performed a tissue microarray (TMA) containing 80 pairs of LUAD tissue and para-tumor tissues. The clinical characteristics of these patients is shown in Table S1, Supplemental Digital Content, http://links.lww.com/MD/M876 and the parameters of the antibody reagents employed in the experiments is shown in Table S2, Supplemental Digital Content, http://links.lww.com/MD/M877. We also calculated the disease stage of these patients based on authoritative international standards and TNM standards.^[26]^ Using Image J, we scanned images from the immunohistological examination^[27]^ to determine the staining area and integrated optical density (IOD). Following this, we used the ratio of the 2 values to calculate the mean optical density (MOD), which is positively associated with the staining intensity of the tissue. The difference in the expression levels between tumor and para-tumor tissues could be specific if expressed as the MOD value. Using the KM curve,we compared the difference of OS rates in the high- and low-MOD groups.

### 2.7. Genetic interaction analysis

GeneMANIA (http://www.genemania.org), a open authoritative website used to construct gene networks, analyze gene lists, and assess specific gene functions,^[28]^ was used to identify neighboring genes of CDCA4/5 from the gene network and for subsequent enrichment analysis.

### 2.8. Functional enrichment analysis

R package “clusterProfiler” and “enrichplot” were installed for gene ontology (GO), Kyoto Encyclopedia of Genes and Genomes (KEGG) pathway enrichment analyses.^[29]^ GO comprises 3 representative categories, namely biological process (BP), cellular component (CC), and molecular function (MF). Gene set enrichment analyses (GSEA) was conducted using GSEA 4.3.2. For the above analyses, the result was considered statistically significant at *P* < .05.

### 2.9. Immune infiltration and therapy analysis

Timer2.0 (http://timer.comp-genomics.org/) is an open authoritative website used to analyze immune association and immune estimation.^[30]^ We used it to explore the correlation among CDCA4/5 expression, tumor purity, and immune cell infiltration. The R package ‘ggExtra’ was installed to analyze the ratio between the tumor mutation burden (TMB) and CDCA4/5 expression. Following this, we retrieved the immune score from TCIA (https://tcia.at/) to analyze the curative effect in the high-and low-expression groups.

### 2.10. Statistical analysis

For the quantitative analysis of our data in our study, we used the Student *t* test when analyzing normally distributed variables and the Wilcoxon rank-sum test when analyzing non-normally distributed variables to analyze differences between groups. Also, we compared survival data using the log-rank test. In the above analysis, the difference was considered statistically significant at *P*-value < .05.

## 3. Results

### 3.1. All CDCAs are predominantly overexpressed in LUAD

**Figure 1.**
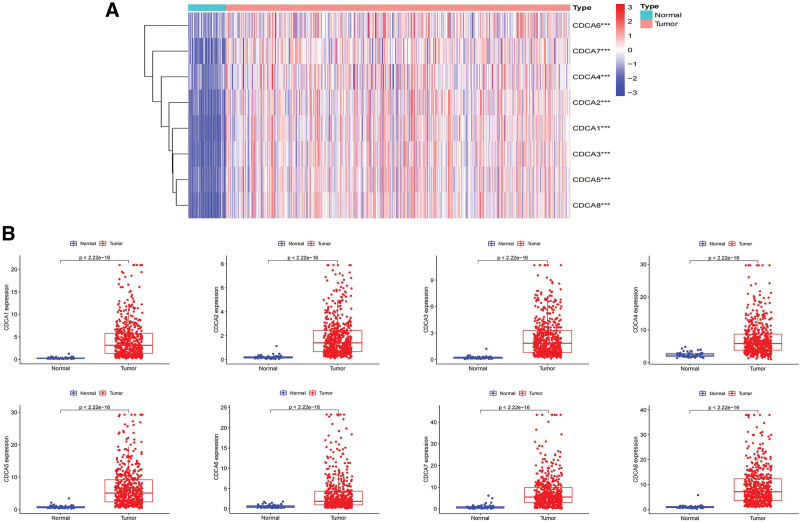
Differential expression analysis of CDCA family members in LUAD. (A) Heat map of CDCAs expression in LUAD. (B) CDCAs were significantly up-regulated in LUAD. CDCA = cell division cycle-associated, LUAD = lung adenocarcinoma.

CDCA expression patterns between LUAD tissues (n = 541) and normal tissues (n = 59) were compared using data from the TCGA database. Our results showed that CDCA1/2/3/4/5/6/7/8 were overexpressed in LUAD tissues than in normal tissues (*P* < .05) (Fig. 1A, B). The details of the results are shown in Table 2.

**Table 2:** Significant changes in CDCA family transcription levels between LUAD and normal tissues.

Gene	Normal mean	Tumor mean	Fold change	*P* value
CDCA1	0.300	4.363	3.860	4.73E-34
CDCA2	0.215	1.891	3.136	1.80E-32
CDCA3	0.235	2.493	3.407	4.25E-34
CDCA4	2.429	7.713	1.562	1.53E-26
CDCA5	0.763	6.680	3.131	8.86E-32
CDCA6	0.710	3.671	2.590	3.47E-21
CDCA7	0.929	7.582	3.030	6.57E-28
CDCA8	1.010	9.124	3.176	2.35E-34

### 3.2. Correlation between CDCA expression patterns and the clinical characteristics

**Figure 2.**
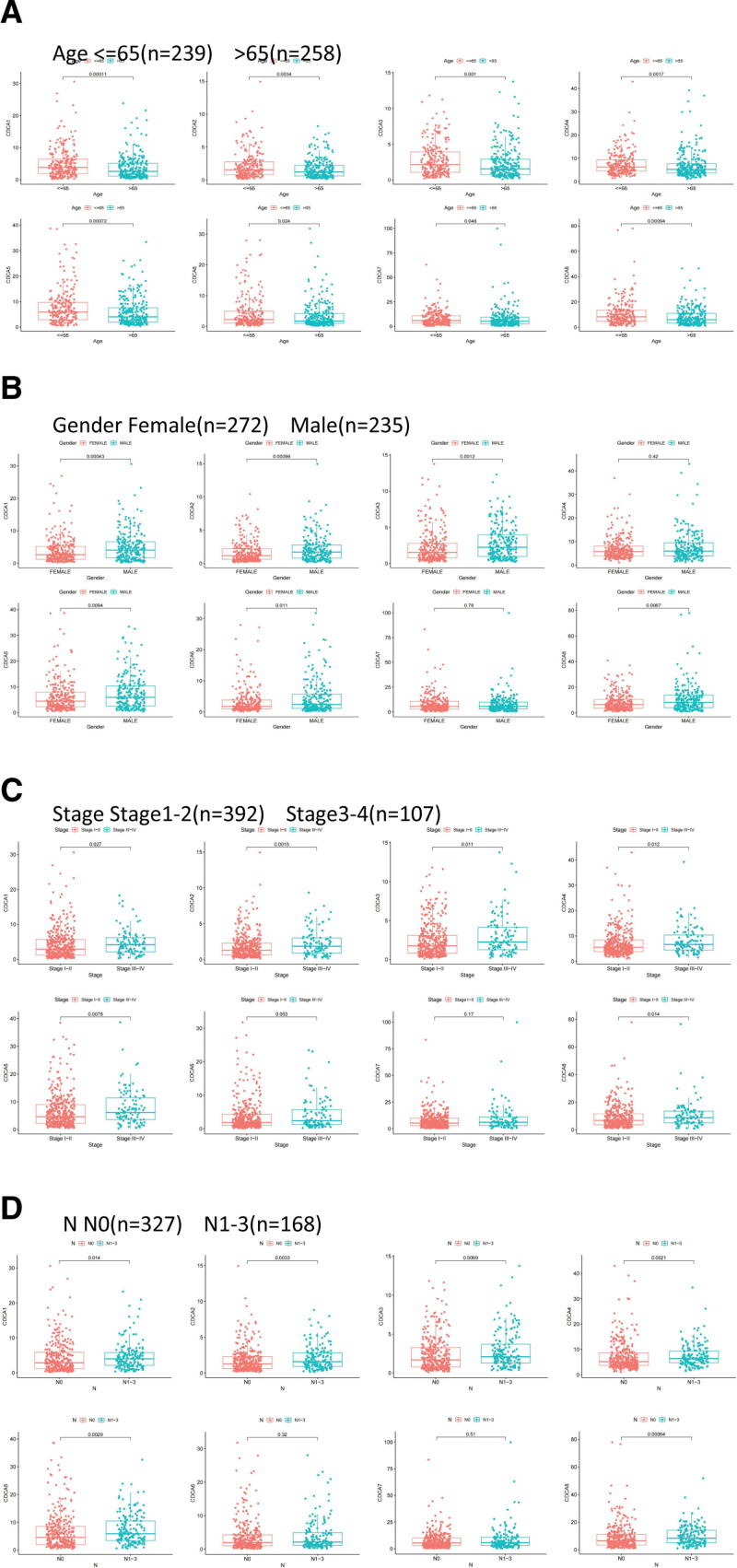
Correlation of CDCA family with clinical factors. (A) Correlation between CDCAs expression and age in LUAD patients. (B) Correlation between CDCAs expression and gender in LUAD patients. (C) Correlation between CDCAs expression and tumor stage in LUAD patients. (D) Correlation between CDCAs expression and N stage in LUAD patients. CDCA = cell division cycle-associated, LUAD = lung adenocarcinoma.

We used R package “ggpubr” to analyze the differences in mRNA expression patterns between groups of patients with LUAD categorized according to their clinical characteristics (age, gender, stage, N). All CDCAs were expressed at higher levels in patients aged 65 years or less than in patients aged above 65 years (Fig. 2A). The expression of CDCA1/2/3/5/6/8 in women was higher than that in men; however, the difference in the expression levels of CDCA4/7 in men and women was not statistically significant (Fig. 2B). The expression levels of CDCA1/2/3/4/5/8 in patients with Stage III–IV were higher than those in patients with Stage I-II. CDCA6/7 expression levels showed no statistically significant difference (Fig. 2C). In the N staging-based groups, the expression levels of CDCA1/2/3/4/5/8 were considerably higher in patients with Stage III–IV than in patients with Stage I–II, whereas the expression levels of CDCA6/7 showed no statistically significant difference (Fig. 2D). The findings from our analysis indicated that CDCA expression is closely associated with clinical characteristics in patients with LUAD.

### 3.3. Alterations in CDCA expression and prognostic value

**Figure 3.**
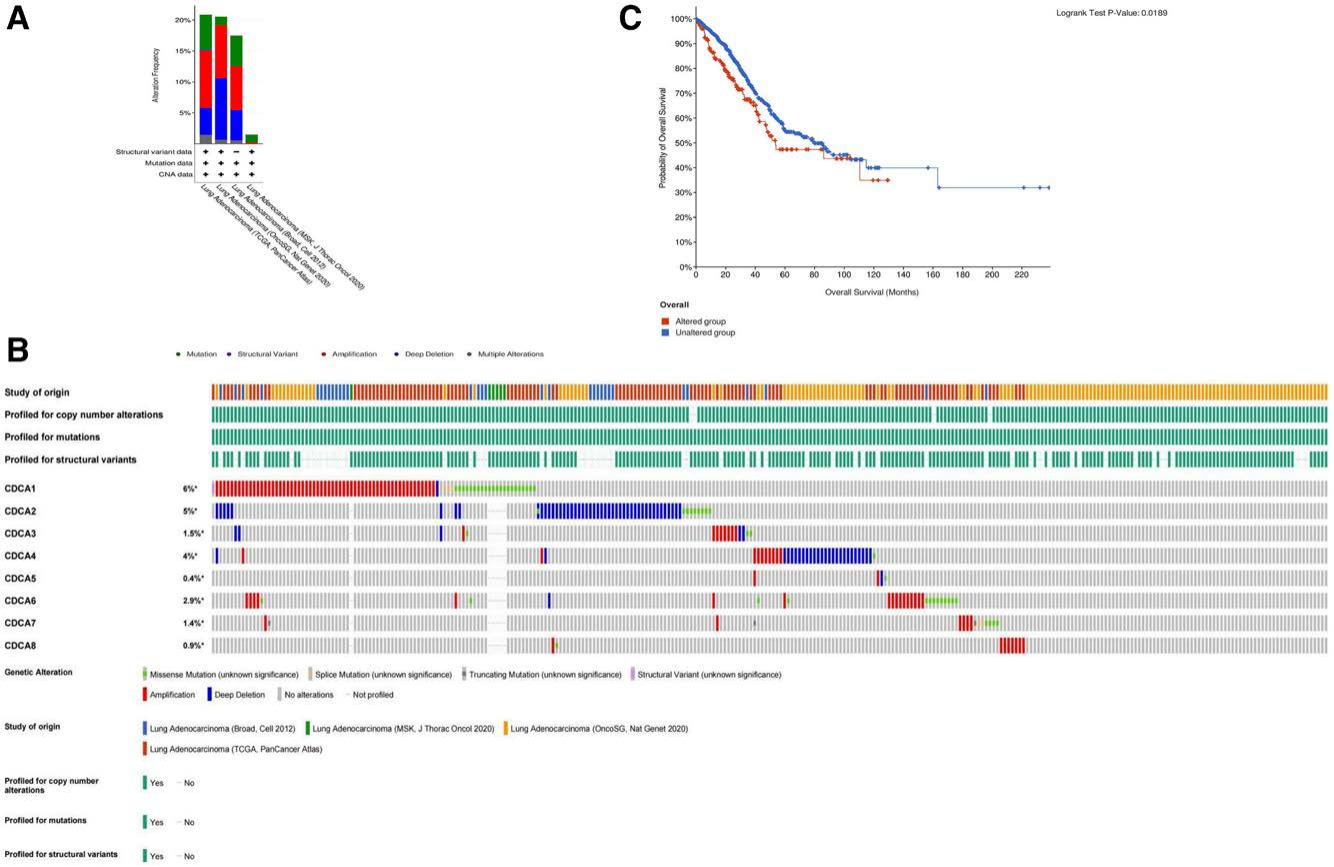
Genetic alterations in CDCA family in patients with LUAD. (A) The genetic alterations of CDCAs in 6 datasets based on TCGA. (LUAD: MSK, J Thorac Oncol 2020, Broad, Cell 2012, OncoSG, Nat Genet 2020, TCGA, PanCancer Atlas) (B) CDCAs alternations in LUAD. (C) Correlation of genetic alterations in CDCAs with OS in LUAD patients. CDCA = cell division cycle-associated, LUAD = lung adenocarcinoma, OS = overall survival.

Alterations in CDCA expression patterns in patients with LUAD were analyzed using data from 4 datasets from the cBioPortal website. Alterations in patients with LUAD included amplifications, mutations, deep deletions, and multiple alterations, ranked from 1.48% (6/406) to 20.85% (118/566) (Fig. 3A). The predominant alteration observed in the 4 datasets was amplifications, followed by deep deletion and then by mutation. The percentages of alterations in CDCAs ranked from 0.4% to 6% in patients with LUAD (CDCA1, 6%; CDCA2, 5%; CDCA3, 1.5%; CDCA4, 4%; CDCA5, 0.4%; CDCA6, 2.9%; CDCA7, 1.4%; CDCA8, 0.9%) (Fig. 3B).

In addition, we investigated the relationship between these alterations in CDCAs and the survival rates. Patients were divided into 2 groups: with alterations and without alterations in CDCAs. KM curve analysis showed that patients with alterations had a poorer overall survival rate than patients without alterations (*P* < .05) (Fig. 3C), which suggests that the prognosis in response to alterations in CDCA genes.

### 3.4. Prognostic value of CDCAs

**Figure 4.**
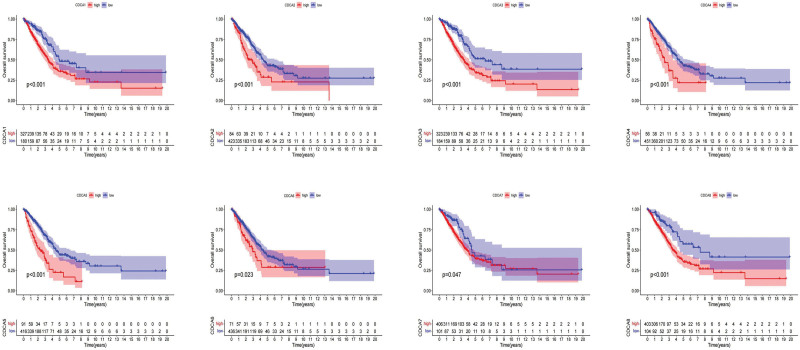
Prognostic value of CDCA family mRNA expression in LUAD. CDCA = cell division cycle-associated, LUAD = lung adenocarcinoma.

Using KM curve analysis, the prognostic values of CDCAs from TCGA database were analyzed. The high expression of all CDCAs was confirmed to be associated with poor overall survival (Fig. 4).

Furthermore, we used univariate and multivariate Cox analyses to identify genes that could be regarded as independent prognostic factors. Based on the results of the univariate Cox analysis, CDCA2/3/4/5/6/8 were identified as risk factors in patients with LUAD (Table 3). Based on the results of the multivariate Cox analysis, both CDCA4 and CDCA5 were potential prognostic biomarkers in patients with LUAD, regardless of other clinical characteristics (Table 3). Considering the abovementioned findings, the prognostic value of CDCAs in LUAD was confirmed, and CDCA4/5 was identified as a potential independent risk factor to predict prognosis.

**Table 3:** Univariate and multivariate COX analysis of CDCAs in the TCGA database.

	UniCox		MultiCox	
	Hazard ratio	*P* value	Hazard ratio	*P* value
CDCA1	1.017 (0.986 − 1.048)	.282		
CDCA2	1.123 (1.050 − 1.201)	<.001		
CDCA3	1.076 (1.018 − 1.137)	.010		
CDCA4	1.046 (1.022 − 1.071)	<.001	1.044 (1.007–1.082)	.019
CDCA5	1.036 (1.016 − 1.056)	<.001	1.039 (1.005–1.073)	.021
CDCA6	1.028 (1.000 − 1.057)	.049		
CDCA7	0.994 (0.977 − 1.012)	.526		
CDCA8	1.015 (1.002 − 1.028)	.022		

### 3.5. Risk score and nomogram based on CDCA4/5 expression

**Figure 5.**
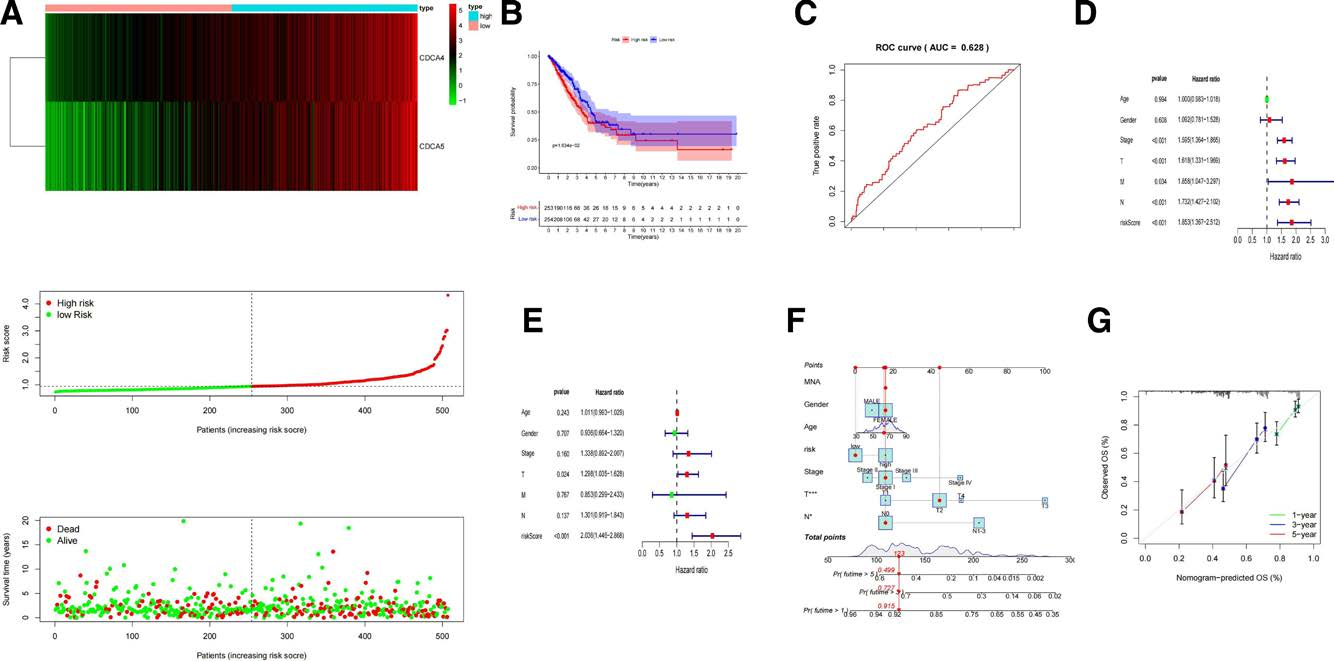
Tumor purity and immune cell infiltration associated with CDCA4/5 expression in patients with LUAD. CDCA = cell division cycle-associated, LUAD = lung adenocarcinoma.

We constructed a CDCA4/5 expression-based risk model. We performed risk scoring using the following formula: (coefficient × CDCA4 expression) + (coefficient × CDCA5 expression). Half of all patients (n = 253) were categorized in the high-risk-score group, and the remaining patients (n = 254) were categorized in the low-risk-score group (Table 4). According to the risk score and prognosis, TCGA samples were classified in specific risk models (Fig. 5A). According to our model, patients in the low-risk group have a better overall survival rate than patients in the high-risk group (Fig. 5B). The ROC curve was used to calculate the accuracy of our model (AUC = 0.628) (Fig. 5C). Furthermore, the univariate Cox analysis showed that age, stage, T, N, and risk score were associated with prognosis (Fig. 5D). The multivariate Cox analysis confirmed that age and risk score could be used as independent prognostic risk factors (Fig. 5E). These results are consistent with our analysis of the prognostic value of CDCA4 and CDCA5. Based on risk score model, we developed a nomogram to predict the survival probability(Fig. 5F). In addition, we adopt the calibration curve to examine the high accuracy of the nomogram to predict the survival duration of patients (Fig. 5G).

**Table 4:** Clinical information of high- and low- risk groups in theTCGA database.

Characteristic (n = 507)		High risk	Low risk
Gender, n (%)	Female	137 (27.0%)	135 (26.6%)
	Male	116 (22.9%)	119 (23.5%)
	Unknown	0 (0%)	0 (0%)
Age, n (%)	<=65	119 (23.5%)	120 (23.7%)
	>65	131 (25.8%)	127 (25.0%)
	Unknown	3 (0.6%)	7 (1.4%)
Pathologic stage, n (%)	Stage I	138 (27.2%)	134 (26.4%)
	Stage II	58 (11.4%)	62 (12.2%)
	Stage III	41 (8.1%)	40 (7.9%)
	Stage IV	11 (2.2%)	15 (3.0%)
	Unknown	5 (1.0%)	3 (0.6%)
T stage, n (%)	T1	100 (19.7%)	69 (13.6%)
	T2	125 (24.7%)	146 (28.8%)
	T3	23 (4.5%)	22 (4.3%)
	T4	3 (0.6%)	16 (3.2%)
	Unknown	2 (0.4%)	1 (0.2%)
N stage, n (%)	N0	159 (31.4%)	168 (33.1%)
	N1	47 (9.3%)	48 (9.5%)
	N2	40 (7.9%)	31 (6.1%)
	N3	0 (%)	2 (0.4%)
	Unknown	7 (1.4%)	5 (1.0%)
M stage, n (%)	M0	165 (32.5%)	173 (34.1%)
	M1	11 (2.2%)	14 (2.8%)
	Unknown	77 (15.2%)	67 (13.2%)
OS event, n (%)	Alive	167 (32.9%)	157 (31.0%)
	Dead	86 (17.0%)	97 (19.1%)
	Unknown	0 (%)	0 (0%)

### 3.6. Immunohistochemical analysis of CDCA4/5

**Figure 6.**
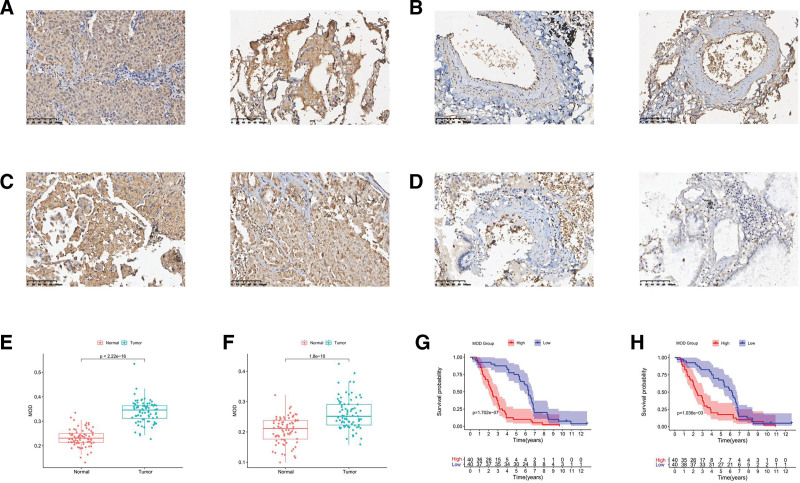
Construction of a CDCA4/5 based risk model in LUAD. (A) Construction of a cox risk proportional regression model for CDCA4/5 in LUAD. (B) The prognosis for the high-risk group divided according to the risk model is significantly poorer than that for the low risk group. (C) Assesing the accuracy of Cox model risk predictions using ROC curves. (D) Univariate and (E) multivariate COX analysis. (F) Integration of risk score and clinical factors to construct nomogram survival predictions. Each factor in the prediction system corresponds to a score, and sum of the scores for all clinical factors corresponds to the total patient score, thus predicting 1 to 3, and 5-year survival rates. (G) Calibration curves for 1 to 3- and 5 year survival predictions in the nomogram prediction system. CDCA = cell division cycle-associated, LUAD = lung adenocarcinoma.

A tissue microarray was constructed for the specific goat antibody staining of CDCA4 and CDCA5 (Fig. 6A–D). We obtained MOD values from the staining result using ImageJ software. By analyzing the MOD values of 80 pairs of tumor and para-tumor tissue, we found that both CDCA4 and CDCA5 are overexpressed in tumors compared with that in normal tissues (*P* < .001) (Fig. 6E, F). In addition, the KM curve revealed that patients with CDCA4 or CDCA5 overexpression had worse prognoses (*P* = 1.702e-07, *P* = 1.036e-03) (Fig. 6G, H). Our experimental results are consistent with the analytical results of the TCGA database. We believe that the high expression of CDCA4 and CDCA5 can be used as independent prognostic markers in LUAD.

### 3.7. Gene-function interaction analysis of CDCA4/5

**Figure 7.**
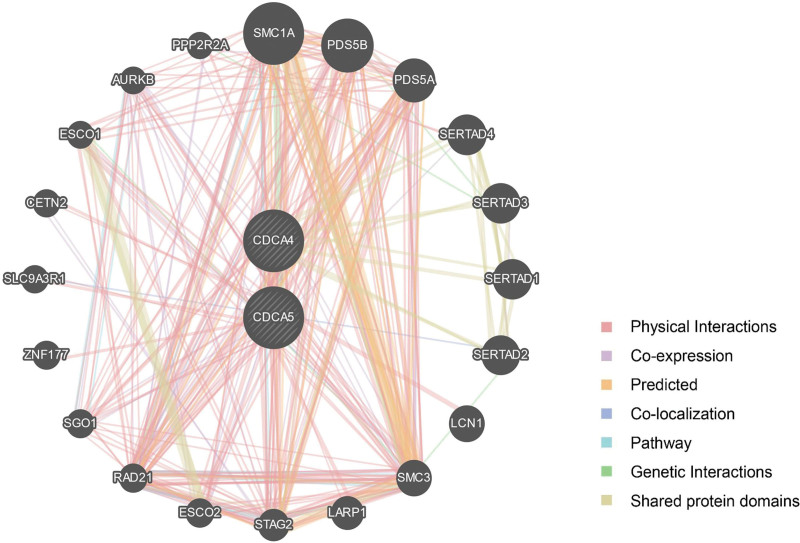
IHC result of CDCA4/5. (A) High expression of CDCA4 in LUAD tissues. (B) Unstained normal tissue. (C) High expression of CDCA5 in LUAD tissues. (D) Unstained normal tissue. (E) Box plot of CDCA4 expression in 80 pairs of paired samples. (F) Box plot of CDCA5 expression in 80 pairs of paired samples. (G) Prognostic analysis of CDCA4 in LUAD specimens. (H) Prognostic analysis of CDCA5 in LUAD specimens. CDCA = cell division cycle-associated, LUAD = lung adenocarcinoma.

To further analyze the genes associated with CDCA4/5 functions, we prepared a gene-related network using the GeneMANIA website and selected 20 genes that most closely interacted with CDCA4/5 (Fig. 7). In CDCA4/5 base network, SMC1A, PDS5B, PDS5A, SERTAD4, SERTAD3, SERTAD1, and SERTAD2 were the 7 genes that showed the most significant interaction with CDCA4/5 (Table 5), with physical interactions being the most significant (77.65%) (Fig. 7). Furthermore, the interacting genes that were functionally related to CDCA4 and CDCA5 were used to investigate the cell cycle for enrichment analysis and identify therapeutic targets in LUAD.

**Table 5:** The 7 most significant genes interacting with CDCA4/5.

Gene	Description	Rank
SMC1A	Structural maintenance of chromosomes 1 A [Source:HGNC Symbol;Acc:HGNC:11111]	1
PDS5B	PDS5 cohesin associated factor B [Source:HGNC Symbol;Acc:HGNC:20418]	2
PDS5A	PDS5 cohesin associated factor A [Source:HGNC Symbol;Acc:HGNC:29088]	3
SERTAD4	SERTA domain containing 4 [Source:HGNC Symbol;Acc:HGNC:25236]	4
SERTAD3	SERTA domain containing 3 [Source:HGNC Symbol;Acc:HGNC:17931]	5
SERTAD1	SERTA domain containing 3 [Source:HGNC Symbol;Acc:HGNC:17932]	6
SERTAD2	SERTA domain containing 3 [Source:HGNC Symbol;Acc:HGNC:30784]	7

### 3.8. Function and pathway of CDCA4/5 in LUAD

GO enrichment analysis was performed to explore the function of CDCA4/5 and similar genes in BP, cellular components (CC), and MF.

**Figure 8.**
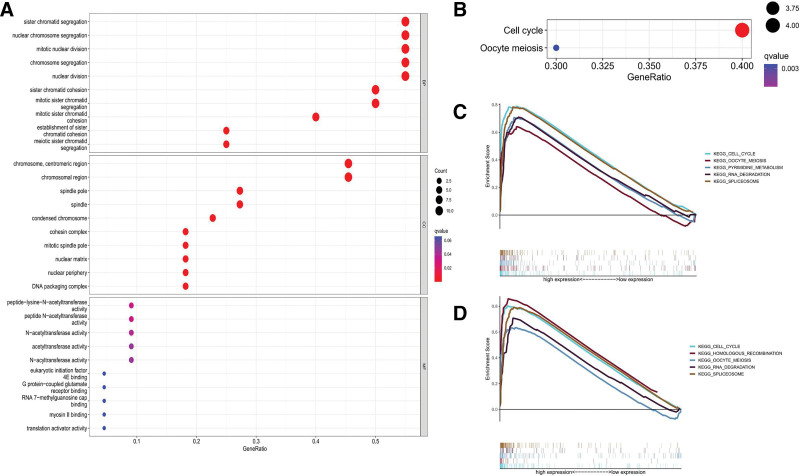
Network of gene interactions associated with CDCA4/5 function.

The functions significantly regulated by CDCA4/5 in BP were sister chromatid segregation, nuclear chromosome segregation, mitotic nuclear division, chromosome segregation, and nuclear division. In CC, chromosome, centromeric region, and chromosomal region were most visibly affected by CDCA4/5 alterations and were predominantly associated with other regulated functions. In MF, peptide − lysine − N − acetyltransferase activity, peptide N − acetyltransferase activity, N − acetyltransferase activity, acetyltransferase activity, and N − acyltransferase activity were most significantly affected (Fig. 8A). In KEGG enrichment analysis, cell cycle and oocyte meiosis were associated with CDCA4/5 (*P* < .05) (Fig. 8B).

From GSEA results, we found that both CDCA4 and CDCA5 are closely associated with the cell cycle (Fig. 8C, D), which is consistent with the results of KEGG analysis and findings from previous studies.

### 3.9. Correlation analysis of CDCA4/5 expression and immune cell infiltration

**Figure 9.**
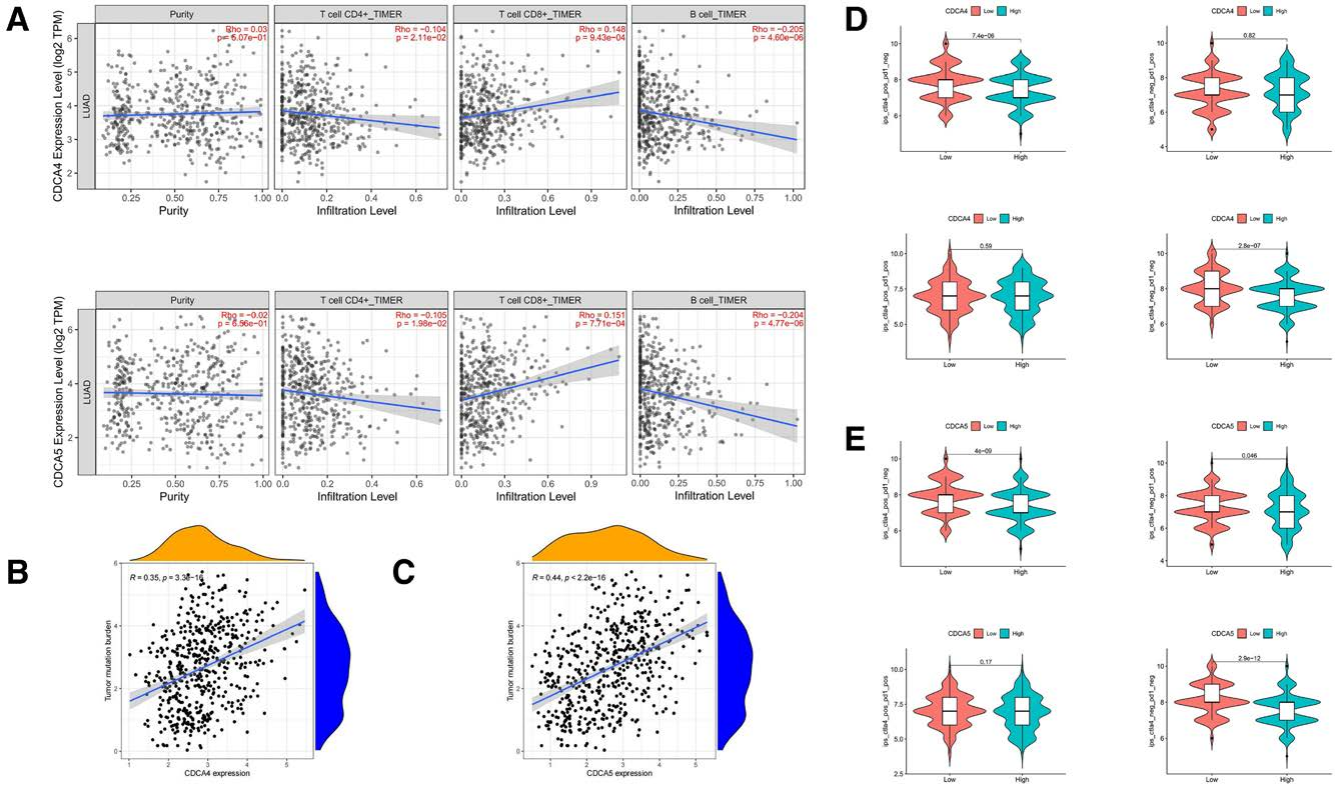
Enrichment of functions and relationships among these functions for CDCA4/5. (A) Enrichment of functions and relationships among these functions in BP,CC and MF for CDCA4/5. (B) KEGG enrichment for CDCA4/5. (C) GSEA enrichment for CDCA4. (D) GSEA enrichment for CDCA5. CDCA = cell division cycle-associated, GSEA = gene set enrichment analyses, KEGG = kyoto encyclopedia of genes and genomes, LUAD = lung adenocarcinoma.

A decline in CD4 + T cell abundance increases the risk of death in lung cancer.^[31]^ The infiltration of CD8 + T cells alone has no prognostic value, but patients with low CD4 + T cell abundance and high CD8 + T cell abundance often have worse prognosis.^[32]^ Another study suggested that infiltrating B cells contribute to antitumor immunity by secreting antibodies and promoting T cell responses, among other ways, which are beneficial to patients.^[33]^ Our findings revealed that the reduced infiltration of CD4 + T cells and increased infiltration of CD8 + T cells and B cells are associated with a worse prognosis when a patient has high expression levels of CDCA4/5 (Fig. 9A). According to our analysis, the TMB is positively correlated with the expression of CDCA4/5 (Fig. 9B, C). This implies that the TMB increases with an increase in CDCA4/5 expression, which indicates a poor prognosis that corresponds with our abovementioned findings. However, an increase in the TMB also indicates the potential immune therapeutic effect in patients with LUAD. We analyzed differences in the immune therapeutic effects in patients who received anti-ctla treatment and anti-pd1 treatment (Fig. 9D, E). Patients with low CDCA4/5 expression showed better results in response to anti-ctla treatment. Patients with low CDCA5 expression showed better results when they received anti-ctla treatment. Patients in the high- and low-expression groups showed no significant difference in response to anti-ctla and anti-pd1 treatment.

## 4. Discussion

Evidence from an increasing number of studies indicates that cell cycle dysregulation is closely associated with cancer development.^[7]^ The cell cycle comprises 4 major phases and the dysfunction in cell cycle phases is a risk factor for cancer progression.^[8]^ CDCA proteins are important components of the cell cycle and play an important role in the occurrence and prognosis of various cancers. However, their diagnostic and prognostic values in LUAD need further investigation.

CDCA1 is a member of the centromere protein complex, which is evolutionarily conserved.^[13]^ Also, CDCA1 is a prognostic marker and a potential immune target in multiple myeloma and prostate cancer.^[34,35]^ Our analysis revealed that CDCA1 is expressed at high levels in LUAD and is associated with poor OS. Furthermore, CDCA1 expression is associated with clinical characteristics like age, gender, stage, and N. CDCA1 was the most frequently altered gene in the CDCAs(6%) and amplification account for a large proportion.

CDCA2 is a diagnostic and prognostic marker for liver cancer^[36,37]^ and promotes hepatocellular carcinoma development via the AKT-mTOR pathway.^[38]^ In this study, CDCA2 was overexpressed in LUAD tissues. In addition, high CDCA2 expression was significantly related to worse OS in LUAD. The expression of CDCA2 was also correlated with age, gender, stage, and N in LUAD. Moreover, deep deletions in CDCA2 had the highest frequency (5%) in LUAD.

Various cancers have been confirmed to be associated with CDCA3 expression, including kidney,^[39]^ bladder,^[40]^ and pancreatic cancer.^[41]^ Also, CDCA3 has been confirmed as a promising target for enhancing the sensitivity of tyrosine kinase inhibitors in patients with TKI-resistant EGFR-mutated NSCLC.^[42]^ In this study, CDCA3 was expressed at higher levels in LUAD tissues than in normal tissues. With respect to survival, higher CDCA3 expression in LUAD was significantly associated with poorer OS in patients. In addition, alterations in the CDCA3 gene was 1.5%, and amplification ranked first.

CDCA4 is a marker for poor prognosis in LUAD,^[43]^ a finding that is consistent with our results. CDCA4 expression increases in most tumors and is associated with poor OS and Disease free survival(DFS).^[44]^ In our study, the expression of CDCA4 was also correlated with the age, stage, and N of patients with LUAD. In addition, alterations in CDCA4 constituted 4% of all alterations, and deep deletion was the predominant genetic alteration. CDCA4 expression is also associated with the infiltration of CD4 + T cells, CD8 + T cells, and B cells (Fig. 9).

CDCA5 and its associated pathways have been widely investigated in cancers, such as the PI3K/AKT/mTOR signaling pathway in breast cancer^[45]^ and the ERK signaling pathway in bladder cancer.^[46]^ Some scholars have found a decrease in the expression of CDK1 in the siCDCA5 cell line,^[46]^ which has been confirmed to be an important protein in the cell cycle. The decrease in CDK1 causes changes in the G2/M-phase cell cycle.^[47]^ From our results, we found that CDCA5 is closely related to the cell cycle in enrichment analysis, which may become a direction for future exploration of CDCA5 promoting the development of lung adenocarcinoma. However, these pathways have not been studied in systemic research for their role in LUAD; we intend to investigate the same in further studies. In this study, the expression of CDCA5 increased visibly in LUAD tissues. In addition, increased CDCA5 expression was significantly associated with a worse OS. CDCA5 expression was correlated with the age, gender, stage, and N of patients with LUAD. Genetic alterations in CDCA5 are considerably low (0.4%), and CDCA5 expression is associated with the infiltration of CD4 + T cells, CD8 + T cells, and B cells (Fig. 5).

From the perspective of immune therapy, the expression of both CDCA4 and CDCA5 is positively correlated with the TMB. In traditional studies, a higher TMB corresponds to a poor prognosis. TMB is defined as the total number of detected somatic gene coding errors, base substitutions, and gene insertion or deletion errors per million bases. However, the higher the TMB, the easier it is for immune cells to monitor and clear the tumor, which also indicates the high feasibility of immune therapy for patients with a high TMB. In further studies, the potential of immune therapy as an efficient auxiliary treatment in LUAD can be investigated.

CDCA6, also known as NUF2, promotes lung adenocarcinoma progression and is associated with a worse prognosis^[48]^; these findings are consistent with our results. In terms of clinical characteristics, CDCA6 is associated with age and gender. Gene alterations in CDCA6 is 2.9% and amplification is most significant alteration.

CDCA7 promotes lymphoma migration and invasion.^[20]^ This finding has great significance in the migration of tumor cells. In our study, CDCA7 is also overexpressed in LUAD tissues, and it is associated with poor OS. However, based on our analysis, the expression of CDCA7 is not associated with clinical characteristics, except age. In terms of alteration, CDCA7 is 1.4% and also closely linked with amplification.

CDCA8 is a biomarker in many cancers, such as liver cancer,^[49,50]^ and ovarian cancer,^[51]^ among others. In our study, CDCA8 was expressed at higher levels in LUAD tissues than in normal tissues, and its expression was associated with poor OS. Furthermore, CDCA8 expression is associated with clinical characteristics such as age, gender, stage, and N. In addition, gene alteration of CDCA3 is 0.9% and amplification is in the first place.

However, this study had some limitations. First, we obtained data primarily from publicly available databases; thus, the accuracy of the data may have affected the reliability of our conclusions. Second, we only conducted a limited number of experiments; more detailed investigation in future studies could help validate our findings.

## 5. Conclusions

Considering the abovementioned findings, CDCAs play important roles in LUAD, and CDCA4/5 can serve as diagnostic and prognostic biomarkers and potential therapeutic targets in LUAD.

## Author contributions

**Formal analysis:** XiangSen Liu, Yi Zhao.

**Funding acquisition:** Kai Yuan.

**Investigation:** XiangSen Liu, Yuchen Shan, Kai Yuan.

**Methodology:** Xudong Zhu.

**Project administration:** Xudong Zhu, ZhaoJia Gao, Kai Yuan.

**Resources:** ZhaoJia Gao, Kai Yuan.

**Software:** Yuchen Shan.

**Supervision:** Kai Yuan.

**Validation:** XiangSen Liu, Kai Yuan.

**Visualization:** XiangSen Liu, ZhaoJia Gao.

**Writing – original draft:** XiangSen Liu.

**Writing – review & editing:** XiangSen Liu, Xudong Zhu.

## Supplementary Material




